# Feature-Based Classification of Mild Cognitive Impairment and Alzheimer’s Disease Based on Optical Coherence Tomographic Angiographic Image

**DOI:** 10.3390/s24165192

**Published:** 2024-08-11

**Authors:** Sarinporn Visitsattapongse, Areerat Maneerat, Adisak Trinavarat, Chatchawan Rattanabannakit, Ekkaphop Morkphrom, Vorapun Senanarong, Varalak Srinonprasert, Dittapong Songsaeng, La-ongsri Atchaneeyasakul, Chuchart Pintavirooj

**Affiliations:** 1 School of Engineering, King Mongkut’s Institute of Technology Ladkrabang, Bangkok 10520, Thailand; sarinporn.vi@kmitl.ac.th (S.V.); 60011210@kmitl.ac.th (A.M.); 2 Department of Ophthalmology, Faculty of Medicine Siriraj Hospital, Mahidol University, Bangkok 10700, Thailand; adisak.tri@mahidol.ac.th; 3 Department of Medicine, Faculty of Medicine Siriraj Hospital, Mahidol University, Bangkok 10700, Thailand; chatchawan.rat@mahidol.ac.th (C.R.); ekkaphop.mor@mahidol.ac.th (E.M.); vorapun.sen@mahidol.ac.th (V.S.); varalak.sri@mahidol.ac.th (V.S.); 4 Department of Radiology, Faculty of Medicine Siriraj Hospital, Mahidol University, Bangkok 10700, Thailand; dittapong.son@mahidol.ac.th

**Keywords:** coherence tomographic angiography, Alzheimer’s disease, machine learning model, OCT-A

## Abstract

Alzheimer’s disease is a type of neurodegenerative disorder that is characterized by the progressive degeneration of brain cells, leading to cognitive decline and memory loss. It is the most common cause of dementia and affects millions of people worldwide. While there is currently no cure for Alzheimer’s disease, early detection and treatment can help to slow the progression of symptoms and improve quality of life. This research presents a diagnostic tool for classifying mild cognitive impairment and Alzheimer’s diseases using feature-based machine learning applied to optical coherence tomographic angiography images (OCT-A). Several features are extracted from the OCT-A image, including vessel density in five sectors, the area of the foveal avascular zone, retinal thickness, and novel features based on the histogram of the range-filtered OCT-A image. To ensure effectiveness for a diverse population, a large local database for our study was collected. The promising results of our study, with the best accuracy of 92.17,% will provide an efficient diagnostic tool for early detection of Alzheimer’s disease.

## 1. Introduction

Alzheimer’s disease (AD) is a progressive neurodegenerative condition and a leading cause of dementia [1]. Globally, approximately 47 million people are affected by AD [2], with projections indicating a rise in this number as the population ages. The pathogenesis of AD is marked by the formation of amyloid plaques and neurofibrillary tangles, composed of aggregated β-amyloid and tau proteins, respectively. Extensive evidence indicates a strong correlation between abnormal β-amyloid accumulation and cerebrovascular pathologies, both contributing to the progression of cognitive decline observed clinically in AD patients [3]. Common clinical manifestations of AD include memory loss, disorientation, behavioral changes, and impaired reasoning and judgment. Prior to the onset of clinical symptoms, individuals may exhibit preclinical AD, characterized by the absence of clinical symptoms, or mild cognitive impairment (MCI), involving mild deficits in cognitive function that do not significantly impact daily activities [4]. Once diagnosed, neuronal damage in AD patients represents an irreversible process. However, early screening and diagnosis before the onset of clinical symptoms are critical for preventing dementia due to AD and for advancing the development of new treatments.

An early diagnosis of the preclinical stage of AD is crucial before the irreversible process begins. A biologic diagnosis of AD can be achieved by detecting biomarkers such as β-amyloid accumulation appearing in cerebrospinal fluid or using positron emission tomography imaging [5,6,7]. However, these methods are often invasive, costly, time-consuming, and limited in their availability. During the last decade, the development of a new diagnostics tool for MCI and/or AD patients has captured researchers’ attention. Feature-based machine learning has been introduced a priori for AD diagnostic tools. Sun et al. [8] introduced a novel learning approach for the identification of AD using a Support Vector Machine (SVM) algorithm. Their method focused on the gray matter density feature derived from T1-weighted Magnetic Resonance Imaging (MRI) scans based on the Ashburner and Friston method [9]. To obtain the feature vectors, T1-weighted images and their corresponding tissue segmentation were first deformed to a brain structure segmentation template using the SVM method. A gray matter tissue density map was then computed by multiplying the spatially normalized gray matter map with the Jacobian determinant. A 1D-dimensional feature vector was extracted from voxels within the brain mask in the template for each subject. 

Sun et al. conducted two types of SVM classification, one using a linear approach and the other incorporating spatial-anatomical information. The proposed method achieved higher accuracy (89.3%) in distinguishing cognitively normal from AD subjects compared to the linear SVM approach (84.6%). Shahbaz et al. [10] used machine learning and data mining techniques, including K-nearest neighbors (K-NN), decision tree (DT), rule induction, Naive Bayes, generalized linear model (GLM) and deep learning algorithm applied the Alzheimer’s Disease Neuroimaging Initiative (ADNI) dataset to classify the different stages of the AD. The original TADPOLE dataset [11] contains 1907 attributes for 1737 participants. Based on the diagnosis, these participants have been divided into five different classes, namely cognitively normal, early MCI, late MCI, subjective memory complaint, and AD. The analysis of results revealed that the generalized linear model outperformed other classifiers, achieving an accuracy of 88.24%.

Tripoliti et al. [12] explored the application of the Random Forest (RF) algorithm to predict Alzheimer’s disease (AD) using both single and multimodal neuroimaging data. Their study involved 41 participants categorized into three groups: 12 AD patients, 14 healthy young controls, and 14 healthy elderly controls. All participants underwent a visual fMRI finger tapping task, and preprocessing of raw structural and functional images included correction of motion artifacts, registration, and normalization. Demographic and behavioral data were integrated with features extracted during preprocessing, such as head motion parameters, volumetric measurements from gray matter (GM), white matter (WM), and cerebrospinal fluid (CSF) segmentation, as well as activation patterns and hemodynamic measures. The researchers utilized these selected features to train an RF classifier consisting of 10 trees and evaluated its performance using 10-fold cross-validation accuracy. The study analyzed two distinct datasets: the first included AD patients, young healthy controls, and elderly healthy controls, while the second focused solely on AD patients and elderly healthy controls. The binary classifiers achieved sensitivity and specificity ranging from 94% to 98%, depending on the subset of features selected. The highest accuracy of 98% was observed in the dataset containing AD patients and elderly controls, demonstrating strong performance in both sensitivity and specificity.

Variations in brain structural elements observed on cerebral magnetic resonance imaging can potentially predict the progression to AD in patients with mild cognitive impairment (MCI). The application of deep learning to cerebral MRI has been extensively researched to develop diagnostic tools for Alzheimer’s disease (AD). Liu et al. [13] proposed a learning framework that integrates multiple deep Convolutional Neural Network (CNN) models to simultaneously segment the hippocampus and classify AD using MRI data. They employed a 3D Densely Connected Convolutional Network (3D DenseNet) to extract features from 3D patches obtained through hippocampal segmentation. The combined features from these models were utilized for disease classification. The method was validated using baseline T1-weighted structural MRI data from 97 AD patients, 233 MCI patients, and 119 normal control subjects, achieving a classification accuracy of 92.5%. Basaia et al. [14] developed a deep learning algorithm based on convolutional neural networks (CNN) to predict individual AD diagnosis and disease progression in patients with MCI using a single cross-sectional brain structural MRI scan. They evaluated their method on 3D T1-weighted images from the ADNI database [15] and their own cohort, achieving classification accuracies up to 75% without requiring prior feature engineering and despite variations in imaging protocols and scanners. They suggested that their algorithm is user-friendly and applicable to new patient data. 

Qiu et al. [16] introduced an innovative deep learning framework that combines a fully convolutional network (FCN) with a traditional multilayer perceptron (MLP) to generate high-resolution visualizations of AD risk and accurately predict disease status. They validated their model across four diverse datasets: ADNI, AIBL, FHS, and NACC. The FCN model used a patch-based training approach with randomly sampled T1-weighted MRI volumes and generated individual-specific probability maps of brain disease. High-risk regions were processed by the MLP for binary disease classification. Additionally, Qiu et al. developed an MLP model based on non-imaging features such as age, gender, and Mini-mental State Examination (MMSE) scores to classify individuals with AD and normal cognition. The multimodal model consistently demonstrated strong performance across datasets, with mean area-under-curve values of 0.996, 0.974, 0.876, and 0.954 for the ADNI, AIBL, FHS, and NACC studies, respectively.

Degeneration of retinal microvasculature is a critical pathological feature associated with the onset and progression of AD and MCI [17,18,19]. However, the intricate nature of retinal microvasculature complicates direct detection using methods like fundus photography and Doppler ultrasonography. Recent advancements in retinal imaging technologies, such as optical coherence tomography angiography (OCT-A), now allow for noninvasive visualization of retinal microvascular networks [20]. OCT-A technology utilizes laser light reflectance from retinal and choroidal vasculature captured in repeated B-scan OCT images from the same retinal location. This technique accurately depicts 3D angiograms of the retinal and choroidal vascular networks by capturing the movements of red blood cells. Several researchers have utilized OCT-A-based features for AD and MCI diagnostic tools. Zabel et al. [21] conducted a direct comparison of vessel density and foveal avascular zone (FAZ) in OCT-A images from 48 eyes of healthy controls and 49 eyes of AD patients, finding significantly greater losses in vascular density in the deep vascular plexus (DVP) and FAZ in AD patients. Li et al. [22] compared nine-sector vessel density in the macular region’s superficial capillary plexus in 29 AD patients and 26 age-matched controls. Li et al. reported noticeable decreases in vessel length density across various macular areas in the AD group compared to controls. Choroid thickness around the macular area and FAZ area also demonstrated degeneration in the AD group compared to controls. Wang et al. [23] studied OCT-A in AD, MCI patients, and controls, focusing on pathologic features such as vessel density in the superficial vascular plexus (SVP) and DVP. Wang et al. concluded that the superficial vascular density was notably reduced in both the AD and MCI patient groups compared to the normal group. Nunes et al. [24] employed fundus images from the OCT data to analyze retinal tissue structure using texture metrics like gray level co-occurrence matrix and dual-tree complex wavelet transform, achieving classification accuracies for healthy controls, AD, and Parkinson’s disease using machine learning (support vector machines). Wisely et al. [25] developed a convolutional neural network (CNN) to detect symptomatic AD using multimodal retinal images and patient data. Their approach combined OCT-A, autofluorescence images, and ophthalmoscopy images with patient demographic data, achieving the best AUC of 0.84 with the ResNet18 deep learning model.

In our research, we developed diagnostic tools to classify MCI and AD using feature-based machine learning applied to an OCT-A image. The salient aspects and/or contributions of this paper are enumerated as follows:

1. Feature-based machine learning will be used as the classifier for MCI and AD. The extracted parameters are vessel density, defined as the percent of an evaluated area where there is circulation in five sectors, the area of FAZ, retinal thickness, and quantitative output from a primary classifier.
2. Novel features based on a histogram are proposed in this research: the full-width at half maximum and central intensity value of the range filter OCT-A image.
3. A large local database will be collected and used in the studies to provide an efficient diagnostic tool based on the geological population.
4. Our proposed single-modality method used only deep-layer OCT-A images and thus required fewer resources compared to the previously proposed multimodality method.

## 2. Materials and Methods

We propose a classification scheme for MCI and AD based on OCT-A technology in which the laser light reflectance of the surface of moving red blood cells is collected through different segmented areas of the eye. The RTVue XR Avanti System with AngioVue software version 2017.1.0.155 (Optovue, Inc. Fremont, CA, USA) [26] with a scanning rate of up to 70,000 A-scans per second is used for data collection. An OCT-A image of 28 AD, 22 MCI patients, and 99 control subjects was collected at the Ophthalmology Department of Siriraj Hospital, Thailand, and approved by the Ethics Review Committee for Research Involving Human Subjects. The age range of AD and MCI patients is between 60–100 years old, while the control subjects are between 50–70 years old. The deep vascular plexus (DVP) OCT-A image is used for feature extraction. The classifiers used in our method are support vector machines due to their interpretability, flexibility, and high-quality results.

### 2.1. Feature Extraction 

**Figure 1:**
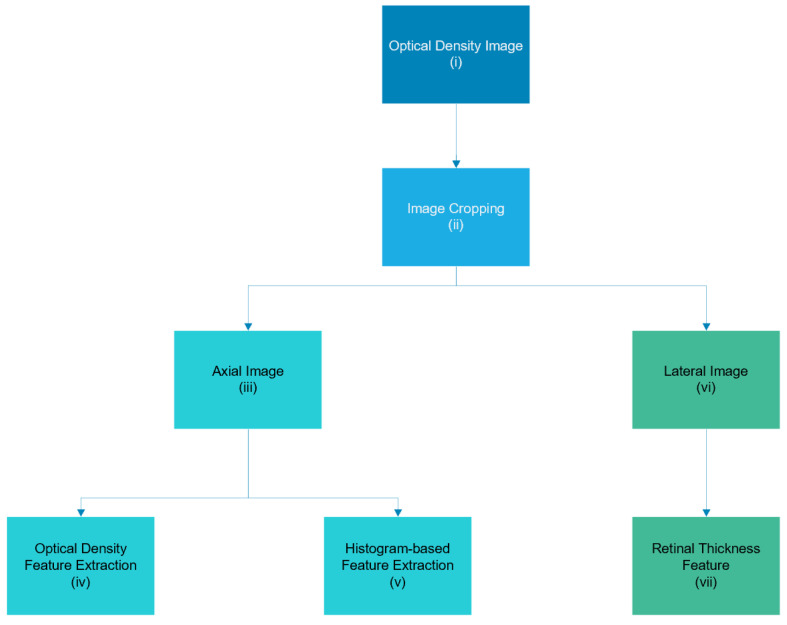
Three distinctive features extracted from the deep-layer OCT-A image, including optical density and FAZ features (iv), histogram-based features (v), and retinal-thickness features (vii).

Three distinctive features are extracted from the deep vascular plexus OCT-A image to classify MCI and AD based on OCT-A images. These include vessel density and FAZ features, histogram-based features, and retinal-thickness features. The optical density and FAZ features, as well as the histogram-based features, are based on using the axial-view OCT-A image, whereas the retinal-thickness features are based on the lateral-view OCT-A image. The feature extraction scheme is shown in Figure 1, and the input of the feature extraction scheme is the raw, deep-layer OCT-A image. Prior to the feature extraction process, the raw image will be automatically cropped to remove the irrelevant components from the images.

#### 2.1.1. Retinal Vessel Density, Reduced Vessel Density Area, and FAZ Feature Extraction 

**Figure 2:**
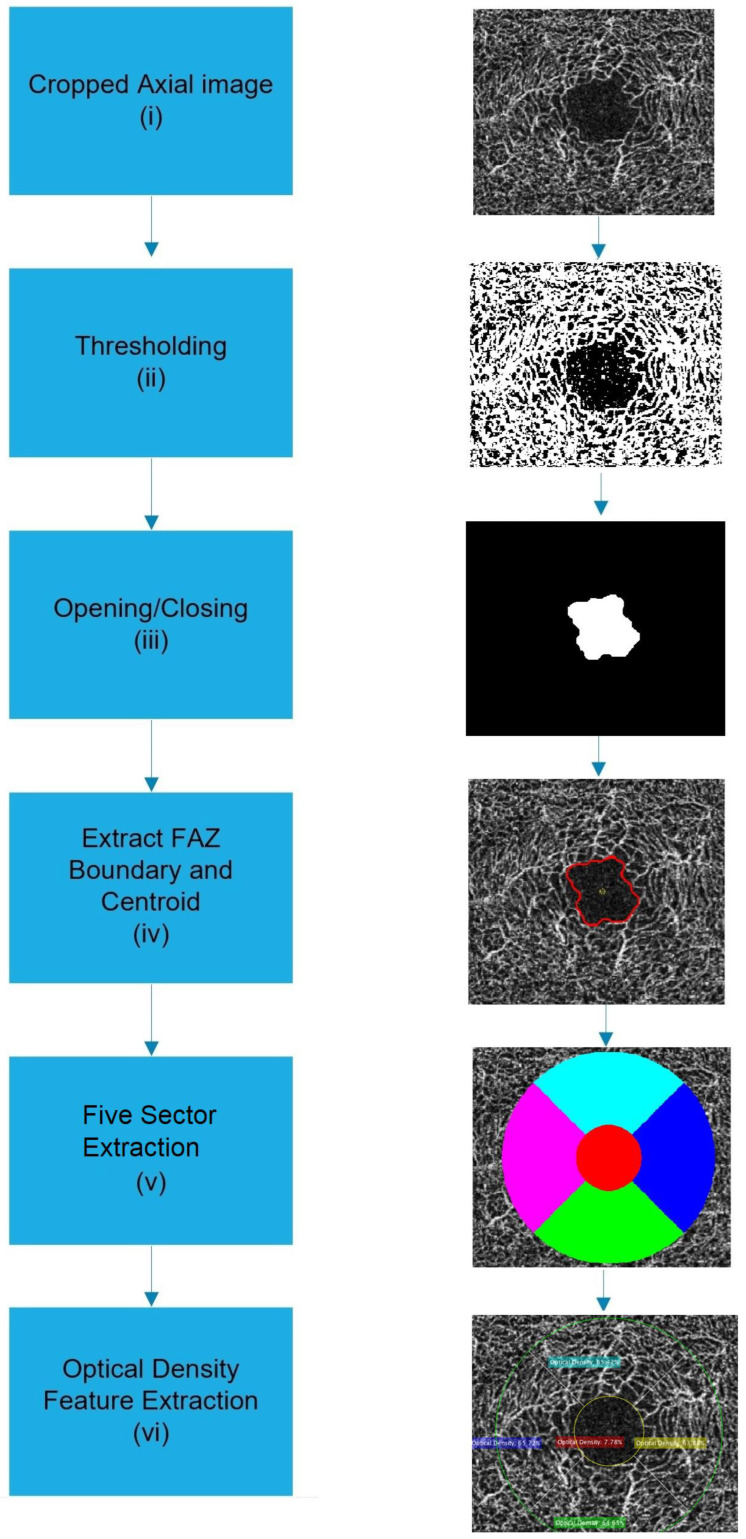
Block diagram of automatic retinal vessel density, five-sector zone and FAZ feature extraction from the OCT-A image. Output of five-sector extraction step (v) is represented in color mapping.

Clinical evidence has shown that the progression of AD results in the degeneration of cerebral blood perfusion [17,18,19]. As the retina is an important part of the central nervous system, the loss of retinal vessel density and increases in the foveal avascular [27], caused by the degenerated nervous system, can be used as a helpful and sensible biomarker to identify AD and MCI diseases. In this research, we developed an automatic algorithm based on OCT-A images for retinal vessel density and FAZ feature extraction. Figure 2 shows the block diagram of the algorithm.
Step (i)Deep-layer OCT-A image around the retinal fovea is captured and used for retinal-vessel density and FAZ feature extraction. The axial-view image will be cropped to remove irrelevant components from the image.Step (ii)The axial-view image is converted to a binary image using the threshold of 48. This threshold is selected to provide the vessel density value close to the reported value of the commercial OCT-A device used to acquire the image data.Step (iii)Opening and closing morphological processes are further applied to remove morphological noise and extract the FAZ. Step (iv)The area of FAZ is computed. FAZ boundary and centroid are extracted. Step (v)The average radius, which is the average distance from the FAZ boundary point to the centroid, is used as the inner radius of a nine-sector ring based on a study of early treatment of diabetic retinopathy. The outer radius is defined as three times the inner radius. Five sector zones can now be defined. Step (vi)Vessel density, defined as the percent pixel of white pixels to the total number of pixels in the five zones, is extracted. 

**Figure 3:**
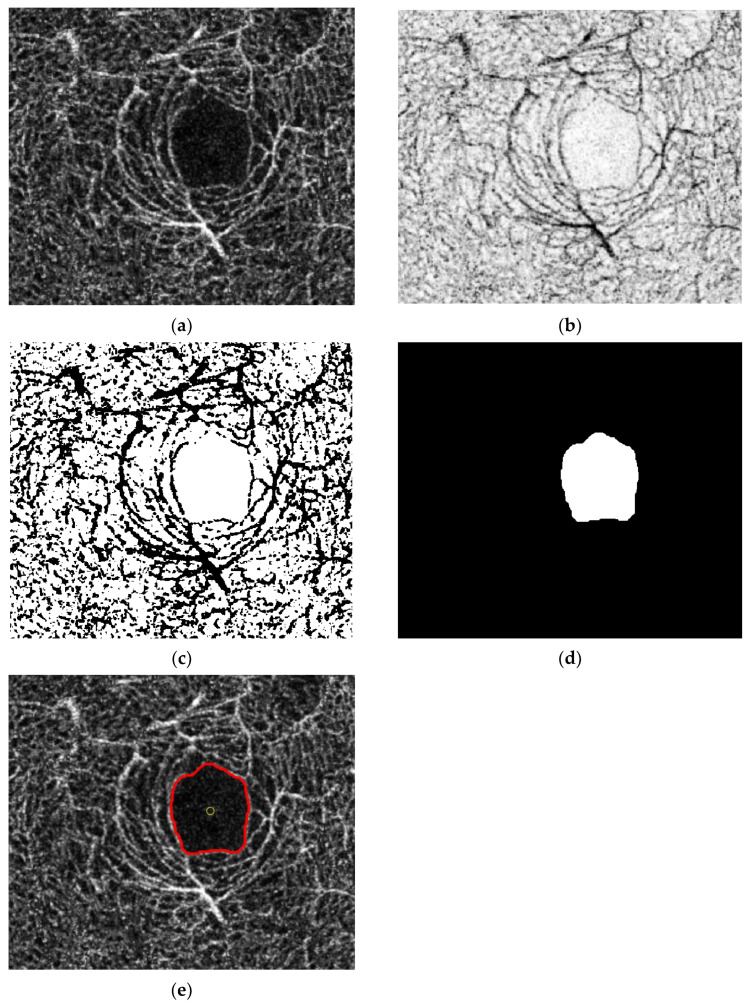
FAZ Feature extraction: (**a**) axial OCTA image; (**b**) negative image; (**c**) thresholding image; (**d**) Opening/Closing image; (**e**) centroid, boundary, and area of FAZ shown in red circle.

The output of the FAZ feature extraction process from the OCT-A image is shown in Figure 3.

To determine the area and centroid of FAZ, we exploit the concept of moment applied to the binary image. The process starts by converting the axial OCT-A image to negative and then applying thresholding shown in Figure 3c. Opening and closing morphological processes are then applied to the binary image to remove pixels related to retinal vessels, leaving only FAZ-related pixels, as shown in Figure 3d. Given a binary image *F*(*x*,*y*) of size *J* and *K*, the moment of the *mth* and *nth* order is defined as [28,29].

(1) $$M(m,n)=\frac{1}{J^{m}K^{n}}\sum_{x=1}^{J}\sum_{y=1}^{K}\left(x{)}^{m}{\left(y\right)}^{n}F(j,k\right)$$

The FAZ area can be determined from *M*(0,0) and centroid $\left(\bar{x},\bar{y}\right)$ can be determined from the ratio of the first-order moment and zero-order moment as:

(2) $$\bar{x}=\frac{M(1,0)}{M(0,0)}$$

(3) $$\bar{y}=\frac{M(0,1)}{M(0,0)}$$

To extract the boundary, we subtract the FAZ binary image from the eroded morphological image [29]. The thickness of the boundary is determined by the structural element, which is a circular shape with a radius of one pixel. The extracted boundary, area, and centroid of FAZ are depicted in Figure 3e. The distance between the boundary point and the centroid is also used as the inner circle of the five-sector zone.

**Figure 4:**
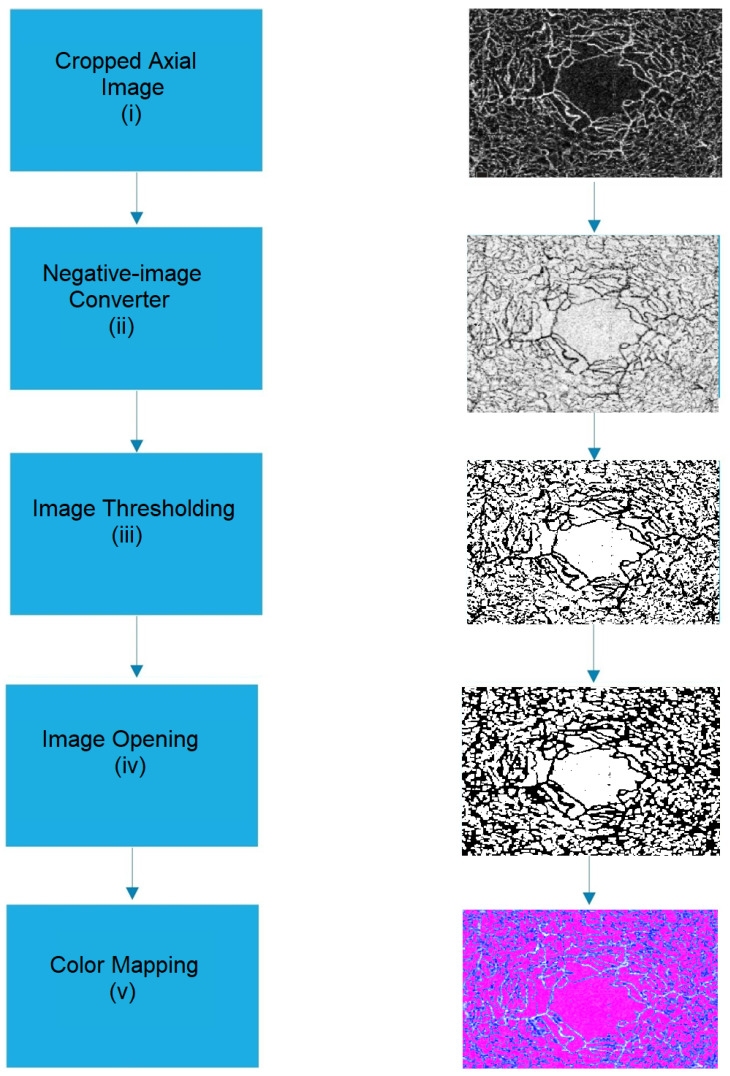
RVDA feature extraction: (i) axial OCT-A image; (ii) negative image; (iii) thresholding image; (iv) opening image; (v) color mapping. RVDA feature is the total magenta area of the step (v) image.

Another feature related to vessel density is reduced vessel density area (RVDA), which is defined as the total area in the axial OCT-A image of which the vessel density is less than some threshold value. Assuming that the patient with AD experiences a degeneration of the central nervous system, the reduced vessel density area should be higher than the normal patient. Figure 4 shows the RVDA feature extraction. The extraction algorithm is described as the following:
Step (i)The deep-layer OCT-A image around the retinal fovea is captured and used for retinal vessel density and FAZ feature extraction. The axial-view image will be cropped to remove irrelevant components from the image. Step (ii)The axial-view image is converted to negative, where the vessel appears as dark pixel intensity.Step (iii)Image thresholding is applied using the threshold of 178. The area of reduced vessel density, where the intensity is above 178, appears as the white area.Step (iv)The opening morphological process is further applied to remove morphological noise and extract the RVDA area.Step (v)Color mapping is applied to provide improved visualization. The RVDA feature is computed by summing RVDA and normalizing to percent.

#### 2.1.2. Histogram-Based Feature Extraction

The research proposes a novel feature based on a histogram. OCT-A retinal images contain bright pixels of retinal vessels on a dark background. The gray-level distribution, or histogram, is normally in a bell shape. For normal patients, the number of bright pixels of retinal vessels is more than the number of dark pixels of the background. However, for patients with AD or MCI, due to the loss of vascular density, the number of bright pixels of retinal vessels is slightly more or less than the number of dark pixels of the background. Hence, histogram parameters, including center intensity or full-width on half-maximum (FWHM) value, can be used as classification features. The histogram-based feature is more prominent when a range filter [30] is applied to the OCT-A image before the histogram application. In the range filter, a window of a determined size, such as 3 × 3, is scanned throughout the image, and the output value at each pixel is replaced with the difference of maximum and minimum (i.e., the range) of intensity values of the pixels in a surrounding region. The algorithm for extraction of the histogram-based feature is as follows:
Step (i)The deep-layer OCT-A image around the retinal fovea is captured and used for retinal vessel density and FAZ feature extraction. The axial-view image will be cropped to remove irrelevant components from the image. Step (ii)The OCT-A image is converted to a grey-scale image. A range filter is applied to the captured OCT-A gray-scale image. Step (iii)The histogram is applied to the filtered image. Step (iv)The peak or center intensity is extracted. Let H(x) be the histogram of the OCT-A gray-scale image, where x is the 256-intensity level. If H_max_(x) is the peak of the histogram, x at the H_max_(x) is the center intensity and is used as a histogram-based feature. Denote the center frequency as FC. Step (v)The FWHM feature is extracted. Define H_half_(x) to be half of H_max_(x). Search for H(x) for H(x) > H_half_(x) where x is between 0 and x_center_. Note it as x_half1_. Search for H(x) for H(x) < H_half_(x) where x is between xcenter and 256. Note it as x_half2_. The FWHM feature is defined as the difference between x_half2_ and x_half1_.

**Figure 5:**
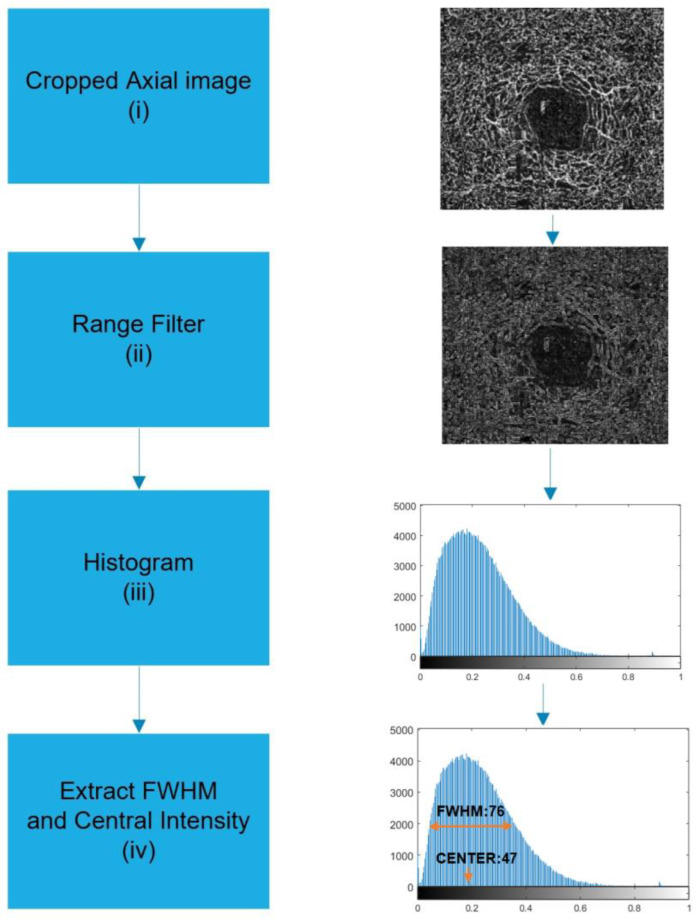
Block diagram of histogram-based feature extraction. (i) Axial OCT-A image; (ii) range filter image; (iii) histogram acquisition; (iv) FWHM and central intensity extraction.

The histogram-based feature step is shown in Figure 5.

#### 2.1.3. Retinal Thickness Feature Extraction 

Retinal thickness, measured by optical coherence tomography, may be used as a biomarker for the presence of neurodegenerative diseases [31]. In this research, the retinal thickness feature is extracted from the lateral OCT-A image and used to classify AD and MCI diseases. The algorithm for retinal thickness feature extraction is described as follows:
Step (i)The deep-layer OCT-A image around the retinal fovea is captured and used for retinal vessel density and FAZ feature extraction. The lateral view image will be cropped to remove irrelevant components from the image. Step (ii)The OCT-A image is converted to a grey-scale image and, finally, a binary image. Step (iii)The opening and closing are further applied to the binary image to remove morphological noise. Step (iv)The boundary is applied to the background zone. The background zone is selected based on the maximum-area output of image labeling.Step (v)The minimal y coordinate of the boundary point is searched. The intensity profile along the minimal point is extracted.Step (vi)Retinal thickness is extracted from the intensity profile by searching for starting and ending points. The starting point is the point where intensity changes from low to high. After the starting point is determined, then the ending point is determined by checking the point where intensity increases more than some threshold. Retinal thickness is the distance between the starting and ending points.

**Figure 6:**
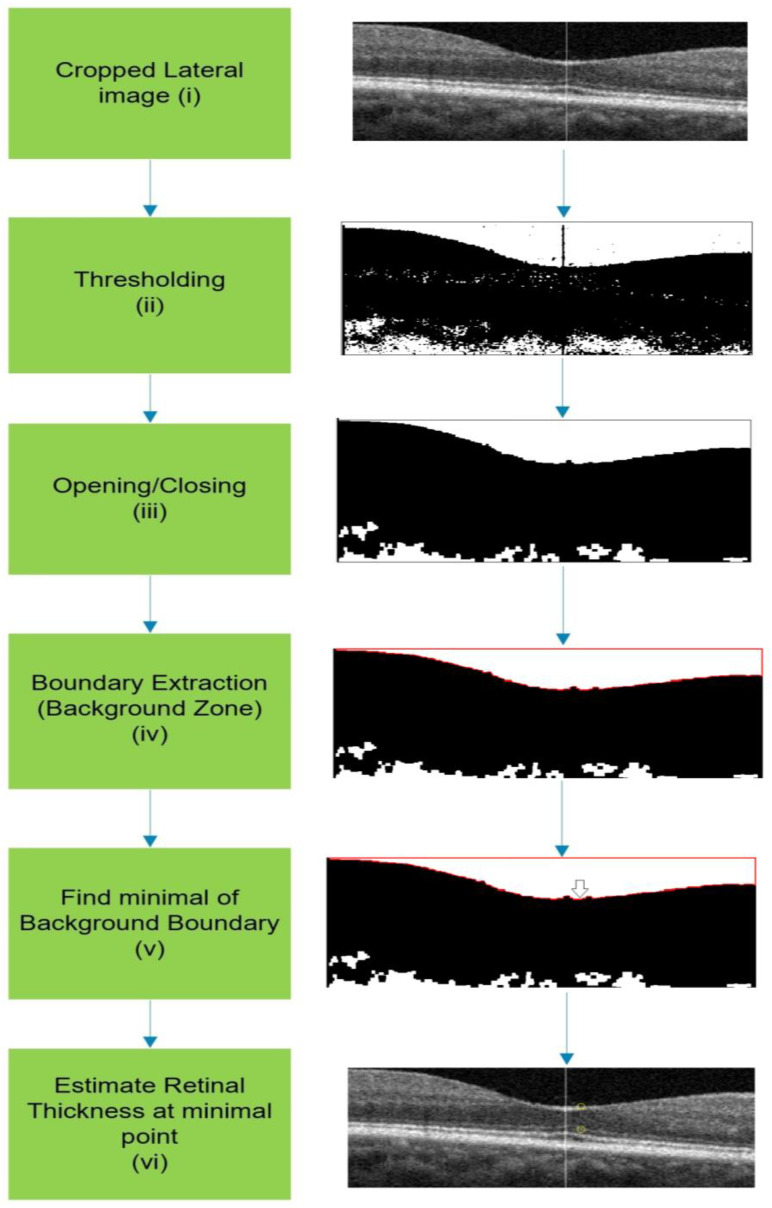
Block diagram of retinal-thickness extraction. (i) Lateral OCT-A image; (ii) thresholding image; (iii) opening and closing image; (iv) background zone boundary extraction; (v) minimal of boundary detection located at white arrow; (v) retinal thickness estimation from the distance between the two yellow circles.

**Figure 7:**
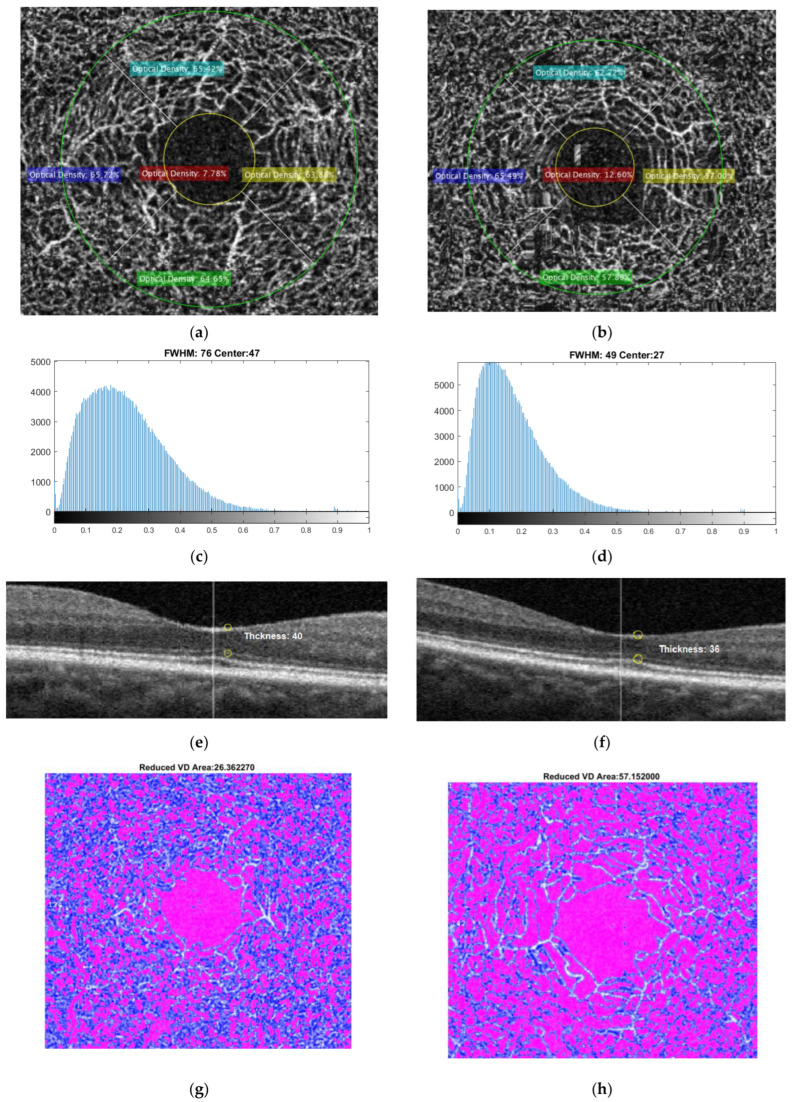
Comparison of all features, including the vessel density-based feature (**a**,**b**), histogram-based feature (**c**,**d**), retinal thickness feature (**e**,**f**), and reduced vessel density (**g**,**h**), from a normal subject (**left column**) and an AD subject (**right column**).

Figure 6 shows the retinal-thickness extraction steps. Figure 7 compares all features, including the vessel density-based feature, histogram-based feature, and retinal thickness feature of a normal subject and an AD subject. 

### 2.2. Classifier 

All features extracted from the OCT-A image, including vessel density-based features, histogram-based features and retinal thickness features, will be applied to unsupervised machine learning classifiers. For the vessel density-based features, the average vessel density of five sectors is used to represent vessel density-based features. A support vector machine (SVM) [32] is used for our classification. SVM is a powerful and popular supervised machine learning algorithm used for classification. The objective of SVM is to find the hyperplane that maximally separates the classes. For non-linearly separable data, SVM applies a kernel function to transform the data into a higher-dimensional space where a linear hyperplane can separate the classes. The algorithm of linear-data SVM is described as the following:
Step (i)Given a hypothesis function,$h_{\theta }\left(x\right)=w_{1}x_{1}+w_{2}x_{2}+\ldots +w_{n}x_{n}-b$ $=w^{T}x-b$(4)The SVM algorithm predicts the classes. One of the classes is identified if $h_{\theta }\left(x\right)=$1, while the other is identified if $h_{\theta }\left(x\right)=-1$. A hyperplane separates the linear data can be written as: 
 $w^{T}x-b=0$
The region bounded within the hyperplane is called the margin.Step (ii)For linear classification, maximize the margin by minimizing the $\left\|w\right\|$ subjecting the constraint $y_{i}\left(w^{T}-b\right)>1$ for all i=0,…,n where $y_{i}$ is the class label 1 or −1${Minimize}_{w,b}\;\;\;{\left\|w\right\|}_{2}^{2}$ subjected to $y_{i}\left(w^{T}-b\right)>1$ for all ∀i ϵ {0,…,n} (5)

### 2.3. Data Collection

The data included in this study was obtained under IRB approval from the Mahidol University and in accord with the principles of the Declaration of Helsinki. Eyes were scanned at the Department of Ophthalmology, Faculty of Medicine, Siriraj Hospital Mahidol University using RTVue XR Avanti System with AngioVue software version 2017.1.0.155 (Optovue, Inc. Fremont, CA, USA) with a 70,000 A-scan-per-second OCT image, frame rate ranging from 256 to 4096 A-scan per frame, 5 µm depth resolution, 15 µm transverse resolution, 2 to 3 mm scanning range, 2 to 12 mm transverse, 840 nm scan beam wavelength, 750 µW exposure power at pupil, 32 × 23 degrees field of view, 3 mm minimum pupil diameter, 22 mm working distance and −15D to +12D motorized focus range. A foveal-centered scan was taken in each eye, resulting in OCT and OCT-A image volumes of size 320 × 320 × 320. Two different OCT-A images of both the deep and superficial retinal capillary networks can be scanned. The superficial OCT-A image visualizes vasculature from the nerve fiber layer and ganglion cell layer, while the deep capillary plexus visualizes both the intermediate and deep retinal vasculature supplying the inner nuclear layer. Both deep and superficial OCT-A images are capable of being scanned with two different scan sizes, including 3 mm × 3 mm and 6 mm × 6 mm. The dataset contains OCT-A images with resolutions of 600 × 600 pixels collected from three groups of subjects, namely normal control group (*N* = 99, age mean ± SD (yrs) = 64 ± 4.49), MCI (*N* = 22, age mean ± SD (yrs) = 69 ± 6.60) and AD (*N* = 28, age mean ± SD (yrs) = 75.4 ± 6.84) group. 

### 2.4. Criterion for Diagnosis of Alzheimer’s Disease/MCI

To exclude normal control subjects, a team of two or more experts is recruited to review history, physical examination, and CT brain or MRI brain examination and to ask questions with a modified Informant Questionnaire on Cognitive Decline in the Elderly (IQCODE) [33].

- If the modified IQCODE score ≤ 3.16 without any history of functional decline or history of other neurological disease, the subject is considered normal.
- If the modified IQCODE score exceeds 3.16, the Montreal Cognitive Assessment (MoCA) [34,35], Barthel index [36], and Lawton scale [37] must be examined to distinguish whether it is MCI or AD. A diagnosis of AD and MCI will be further evaluated according to the *Diagnostic and Statistical Manual of Mental Disorders, Fifth Edition (DSM-5)* [38].

## 3. Experiments and Results 

We have conducted the experiment to perform two binary classifications between normal and AD and between normal and MCI on a 4GB-RAM i7 Intel professor notebook. Two different SVD classifiers are used, including an SVD classifier with one and two features. The SVD classifier with one feature will use the single feature proposed, e.g., the vessel density feature (VD), reduced vessel density area (RVDA), full-width at half-maximum feature (FWHM), central frequency (FC), and retinal thickness (RT). The SVD classifier with two features will optimize from two single features. In total, there are $\left(\begin{array}{c} n\\ 2 \end{array}\right)$ combination classifiers where *n* is a number of single features, which is 5. Different SVD Kernels are also optimized, including polynomial kernel, Gaussian kernel, Gaussian radial basis function (RBF), Laplace RBF kernel, hyperbolic tangent kernel, sigmoid kernel, Bessel function of the first kind kernel, and ANOVA radial basis kernel. The result from the best kernel will be reported for each classifier. A ROC curve will be provided to evaluate the performance of each classifier and confusion matrix. From the derived confusion matrix, the following binary classification metrics will be calculated:

(6) $$Accuracy=\frac{TP+TN}{TP+FP+TN+FN}$$

(7) $$Specificity=\frac{TN}{FP+TN}$$

(8) $$Sensitivity=\frac{TP}{TP+FP}$$

(9) $$Recall=\frac{TP}{TP+FN}$$

where TP, TN, FP and FN are true position, true negative, false position, and false negative, respectively. 

**Figure 8:**
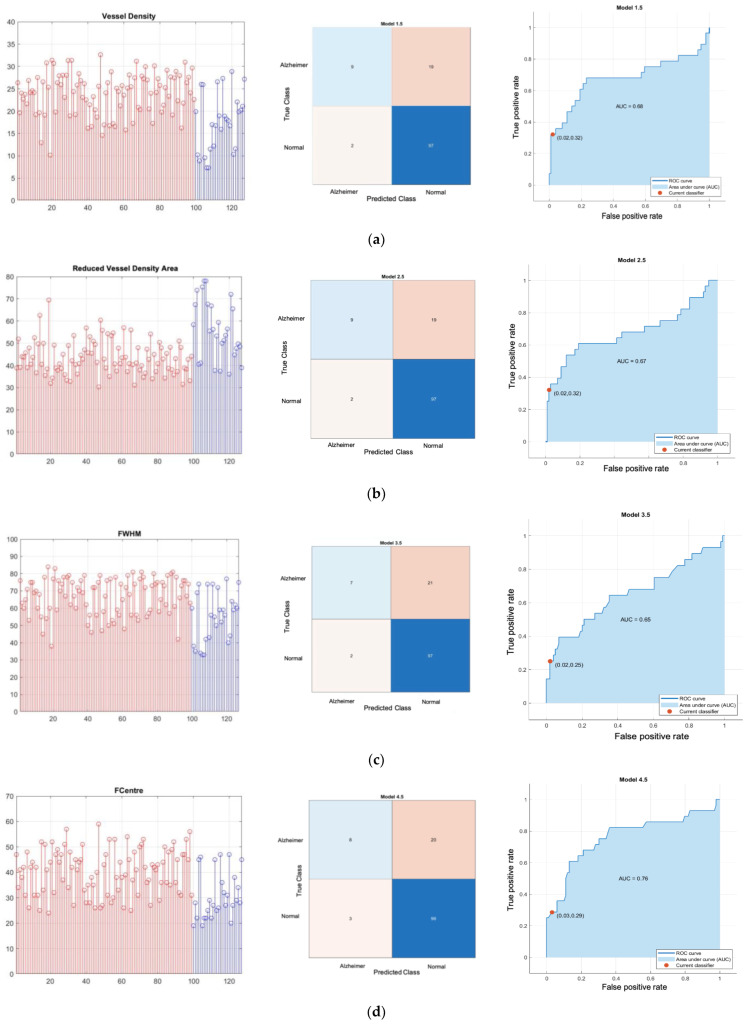

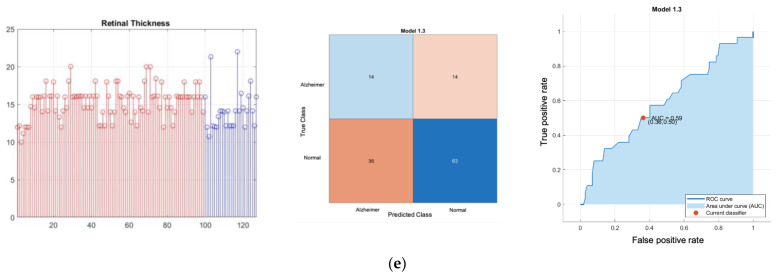
Normal versus Alzheimer’s disease SVD 1D classifier; (**a**) VD feature linear-kernel SVD classifier; (**b**) RVDA feature linear-kernel SVD classifier; (**c**) FWHM feature linear-kernel SVD classifier; (**d**) FC feature linear-kernel SVD classifier; (**e**) RT feature linear-kernel SVD classifier. **Left**: scatter plot (red is control normal group; blue is Alzheimer’s group); **middle**: confusion matrix; **right**: ROC. Note that the color intensity of confusion matrix in middle column is automatically varied with the number.

**Figure 9:**
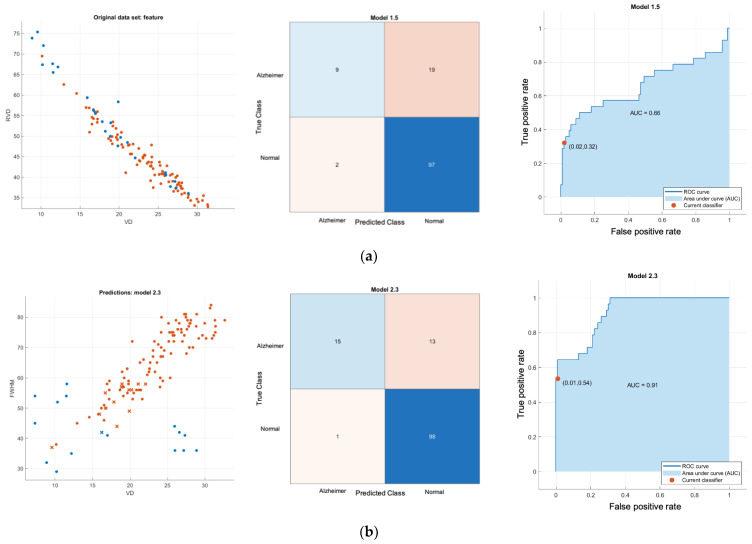

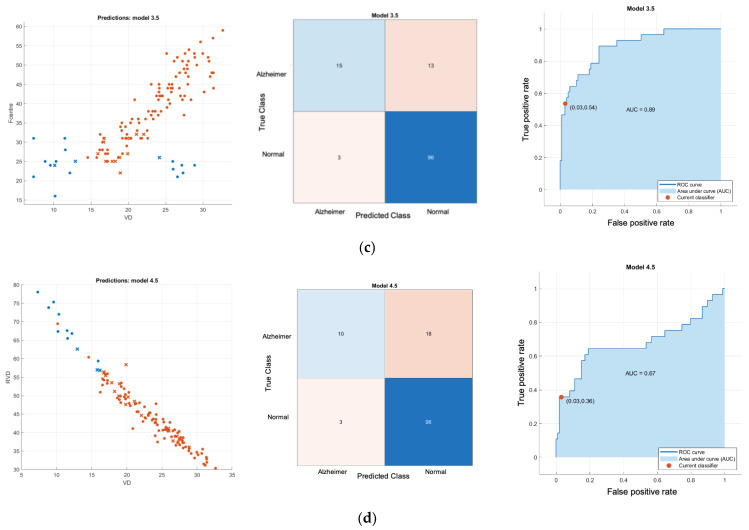
Normal versus Alzheimer’s disease SVD 2D classifier; (**a**) linear-kernel VD-RVDA feature SVD classifier; (**b**) Gaussian-kernel VD-FWHM feature SVD classifier; (**c**) Gaussian-kernel VD-FC feature SVD classifier; (**d**) linear-kernel VD-RT feature SVD classifier. **Left**: scatter plot; **middle**: confusion matrix; **right**: ROC. Note that for scatter plot in the first column blue circle denoted as correct classified class of Alzheimer’s disease (True negative), blue x denoted as incorrect classified class of Alzheimer’s disease (false negative), red circle denoted as correct classified class of normal (True positive), red x denoted as incorrect classified class of normal (false positive).

**Figure 10:**
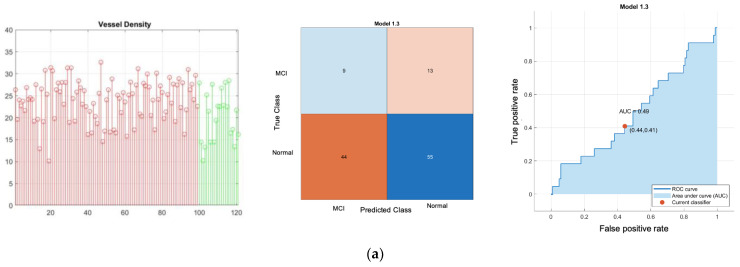

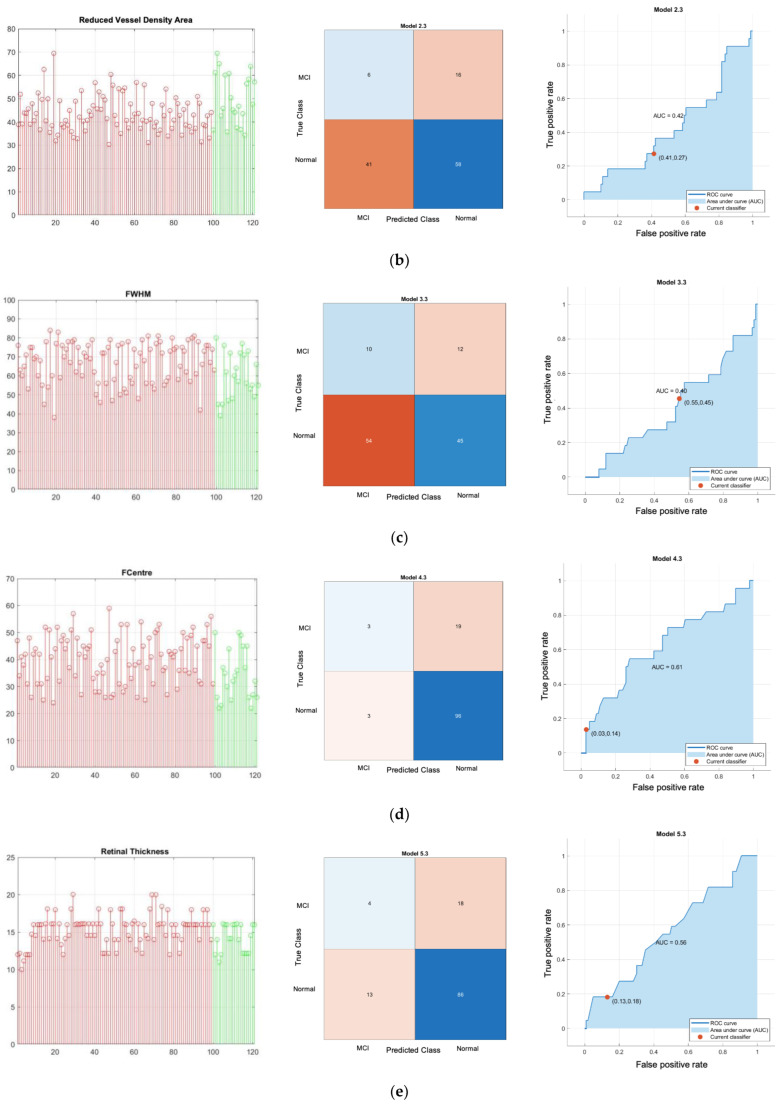
Normal versus MCI SVD 1D classifier; (**a**) VD feature linear-kernel SVD classifier; (**b**) RVDA feature linear-kernel SVD classifier; (**c**) FWHM feature linear-kernel SVD classifier; (**d**) FC feature linear-kernel SVD classifier; (**e**) RT feature linear-kernel SVD Classifier. **Left**: scatter plot (red is control norma group; green is MCI group); **middle**: confusion matrix; **right**: ROC.

**Figure 11:**
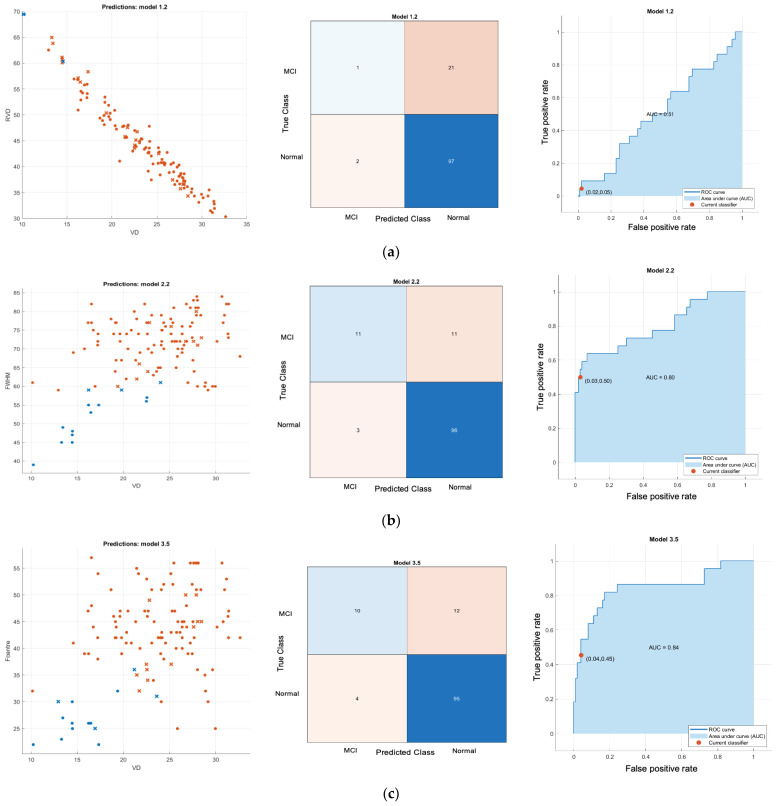

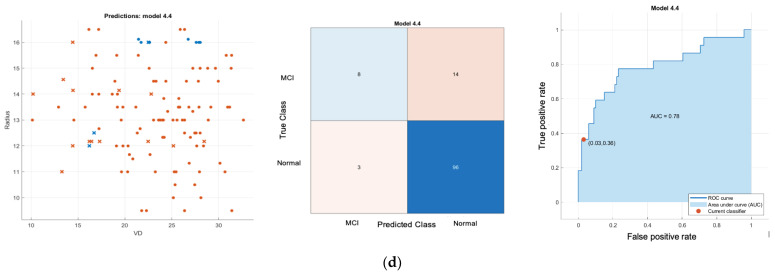
Normal versus MCI SVD 2D classifier; (**a**) Gaussian-kernel VD-RVDA feature SVD classifier; (**b**) quadratic kernel VD-FWHM feature SVD classifier (**c**) Gaussian-kernel VD-FC feature SVD classifier; (**d**) Gaussian-kernel VD-RT feature SVD classifier. **Left**: scatter plot; **middle**: confusion matrix; **right**: ROC. Note that for scatter plot in the first column blue circle denoted as correct classified class of MCI (True negative), blue x denoted as incorrect classified class of MCI (false negative), red circle denoted as correct classified class of normal (True positive), red x denoted as incorrect classified class of normal (false positive).

Figure 8 shows results of normal versus Alzheimer’s disease SVD 1D classifier where plot of feature, confusion matrix and receiver operating characteristic (ROC) plot with area under the curve (AUC) are depicted on the left, middle and right respectively. Figure 9 shows results of normal versus Alzheimer’s disease SVD 2D classifier. Similarly, plot of feature, confusion matrix and receiver operating characteristic (ROC) plot with area under the curve (AUC) are depicted on the left, middle and right respectively. Figure 10 and Figure 11 show classifier result of normal versus MCI of 1D and 2D features respectively. 

**Figure 12:**
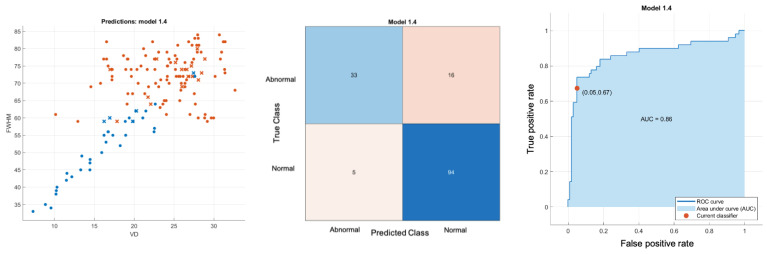
Normal versus combined MCI + AD Gaussian kernel SVD 2D classifier. **Left**: scatter plot; **middle**: confusion matrix; **right**: ROC.

All Classifier metrics are computed and tabulated in Table 1. From Table 1, the best model for both normal versus MCI and normal versus AD is a VD-FWHM 2D feature SVD classifier, which can achieve 89.68% and 92.17% accuracy, respectively. To investigate further, we conducted another binary classifier for normal and combined AD with MCI using the VD-FWHM 2D feature SVD classifier. The output is shown in Figure 12 and the last row of Table 1. 

## 4. Discussion

Although a feature-based SVD classification result of MCI and AD based on OCT-A image is satisfactory, there are certain issues that need to be addressed for the completeness of the research.

1. Although the three-class classification of normal, MCI, and AD is available, we elect to conduct two binary class classifications, i.e., normal versus MCI and normal versus AD, as there is an undominant difference between the MCI and AD features and results in poor performance classification metrics.
2. The retinal thickness unit is in pixels, and the range is between 10 and 20 pixels. To convert to physical units, a conversion of 30 mm/600 pixels can be applied, resulting in the physical unit range of 0.05–1.0 mm. Due to the narrow range of retinal thickness, the feature looks more discrete, as seen in Figure 8e.
3. Compared among all SVD classifiers, the Gaussian-Kernel FWHM-VD 2D feature SVD classifier of normal vs. AD performs superior to the others with an AUC of 0.89 and accuracy, sensitivity, specificity, and recall values of 92.17%, 88.29%, 53.57% and 93.75%, respectively. Compared with Nunes et al. [24] and Wisely et al. [35], our proposed method not only uses fewer resources (only OCT-A image) but also performs better. The accuracy of Nunes et al. was reported at 82.2%, whereas the AUC of Wisely et al. was reported at 0.836. The best normal vs. MCI binary classifier is also Gaussian-Kernel FHWM-VD 2D feature with accuracy, sensitivity, specificity, recall, and AUC values of 89.69%, 89.72%, 50.00%, 73.33%, and 0.84, respectively. For the 1D feature normal-AD binary classifier, RVDA provides the best performance with accuracy, sensitivity, specificity, recall, and ROC values of 85.38%, 83.62%, 32.14%, 81.82% and 0.61, respectively. For the 1D feature normal-MCI binary classifier, RVDA provides the best performance with accuracy, sensitivity, specificity, recall, and AUC values of 89.34%, 88.89%, 45.45%, 76.92%, and 0.40, respectively.
4. Our proposed classifier seems to maximize the specificity, i.e., maximize true negative. The best FWHM-VD classifier for normal vs. AD and normal vs. MCI provides a sensitivity of 88.29% and 89.28%, respectively. The diagnosis for normal subjects will provide an error of less than 12%. However, for the best FWHM-VD classifier for normal vs. AD, the number of true negatives of 15 and false negatives of 13, i.e., the sensitivity of only 50%, possibly degrades the performance. As a result, a machine-learning model might be used for screening purposes. Conventional diagnosis for AD and MCI should be recommended, especially for AD and MCI classifying cases.
5. Overall, our classifier for normal vs. AD and normal vs. MCI can yield an accuracy of 92.17% and 89.69%, respectively, despite the low-sensitivity metrics. This is due to the unbalanced data number between normal, AD, and MCI. The number of normal is about four times as much as the number of AD or MCI. Future investigations are to be explored to collect more cases of AD and MCI.
6. Combined case AD with MCI has been tested for binary classification against normal. The result demonstrates that, with the FWHM-VD classifier, the classification metrics demonstrate promising results with accuracy, sensitivity, specificity, recall, and AUC values of 96.51%, 85.45%, 67.35%, 86.84%, and 0.86, respectively, and with almost a twenty percent improvement in sensitivity.

## 5. Conclusions

Alzheimer’s disease (AD) leads to nerve cell death and tissue loss throughout the brain, including the retina area. The changes in the retina are associated with degeneration and loss of neurons, reduction of the retinal nerve fibers, increase in optic disc area, retinal vascular tortuosity, and retina vascular thinning. This paper explores the possibility of applying machine learning to classify AD. Various features are extracted from the optical coherence tomographic angiography images (OCT-A), including well-known and novel features. The well-known features are vessel density (VD) and retinal thickness (RT). The novel features proposed in this paper are reduced vessel density area (RVDA), histogram full-width at half-maximum (FWHM) and central frequency (FC). A support vector machine (SVM) was used for two binary classifiers: normal versus Alzheimer’s disease and normal versus mild cognitive impairment (MCI). One feature and two-feature models have been optimized to find the best classifier. The best SVD classifier of normal vs. AD performs is the FWHM-VD feature with an ROC of 0.89, accuracy, specificity, sensitivity, and recall values of 92.17%, 88.29%, 53.57%, and 93.75%, respectively. The best normal vs. MCI binary classifier is also the FHWM-VD 2D feature with accuracy, specificity, sensitivity, recall, and ROC values of 89.69%, 89.72%, 50.00%, 73.33% and 0.84, respectively. Although the proposed model can achieve the best accuracy of 92.17%, the specificity does not provide satisfactory results. This is due to the unbalanced data number between normal versus AD and/or MCI. Finally, the combined AD and MCI case versus normal classifier was conducted, achieving an accuracy of 96.7%.

## Figures and Tables

**Table 1:** Binary classification metrics of SVD classifier between normal vs. AD and normal vs. MCI.

Case	Feature(s)	Accuracy	Sensitivity	Specificity	Recall
Normal-AD	VD	85.38	83.62	32.14	81.82
Normal-AD	RVDA	85.38	83.62	32.14	81.82
Normal-AD	FWHM	83.38	82.20	25.00	77.78
Normal-AD	FC	84.19	82.76	28.57	80.00
Normal-AD	RD	63.61	81.82	50.00	28.00
Normal-AD	RVDA-VD	85.38	83.62	32.14	81.82
Normal-AD *	FWHM-VD	92.17	88.29	53.57	93.75
Normal-AD	FC-VD	90.59	88.07	53.57	83.33
Normal-AD	RD-VD	85.59	84.21	35.71	76.92
Normal-MCI	VD	54.45	80.88	40.91	16.98
Normal-MCI	RVDA	53.93	78.38	27.27	12.77
Normal-MCI	FWHM	89.34	88.89	45.45	76.92
Normal-MCI	FC	82.34	83.48	13.64	50.00
Normal-MCI	RD	75.07	82.69	18.18	23.53
Normal-MCI	RVDA-VD	80.51	82.20	4.55	25.00
Normal-MCI *	FWHM-VD	89.69	89.72	50.00	73.33
Normal-MCI	FC-VD	88.69	88.89	45.45	71.43
Normal-MCI	RD-VD	87.34	87.27	36.36	72.73
Normal-Combined AD + MCI	FWHM-VD	96.51	85.45	67.35	86.84

* Best metrics.

## Data Availability

The data that support the findings of this study are available on request from the corresponding author (L.A.). The data is not publicly available due to privacy or ethical restrictions.
